# The regulatory mechanisms of SARS-CoV-2 N protein helicase and its annealing activity

**DOI:** 10.1016/j.isci.2025.113983

**Published:** 2025-11-13

**Authors:** Bo Zhang, Peng Zhou, Zhaoling Lan, Chaoshao Yang, Jida Li, Yi Zhang, Long Gao, Hongyi Wang, Cai Meng, Shizheng Wei, Chenglang Ruan, Yangxue Dai, Yan Xie, Yang Liu

**Affiliations:** 1 College of Basic Medicine, Zunyi Medical University, Zunyi, Guizhou Province 563000, China; 2 School of Public Health, Zunyi Medical University, Zunyi, Guizhou Province 563000, China; 3 Guizhou Provincial Doctoral Innovation Station, Zunyi Center for Disease Control and Prevention, Zunyi, Guizhou Province 563000, China

**Keywords:** Biochemistry, Virology, Biophysics

## Abstract

The nucleocapsid (N) protein of SARS-CoV-2 performs multiple functions essential for viral replication and host adaptation. Here, we identify ionic strength as a critical determinant of its dual nucleic acid-manipulating activities. Under low-salt conditions (≤100 mM NaCl), SARS-CoV-2 N primarily promotes strand annealing, whereas at high ionic strength (≥300 mM NaCl), its helicase activity is additively enhanced. SARS-CoV-2 N also preferentially acts on DNA rather than RNA substrates and modulates the helicase activity of Nsp13 in an ion-dependent manner—inhibiting it under low-salt conditions but enhancing it under high-salt conditions. These findings reveal an ion concentration-dependent regulatory mechanism that enables SARS-CoV-2 N to dynamically switch between distinct biochemical states, thereby supporting viral adaptability and providing new insights into antiviral development.

## Highlights

• SARS-CoV-2 N preferentially unwinds and anneals DNA substrates
• The function of SARS-CoV-2 N is modulated by salt concentration and its own abundance
• SARS-CoV-2 N inhibits Nsp13 helicase activity under low-salt conditions
• SARS-CoV-2 N and Nsp13 additively enhance helicase activity under high-salt conditions

## Graphical abstract

**Figure:**
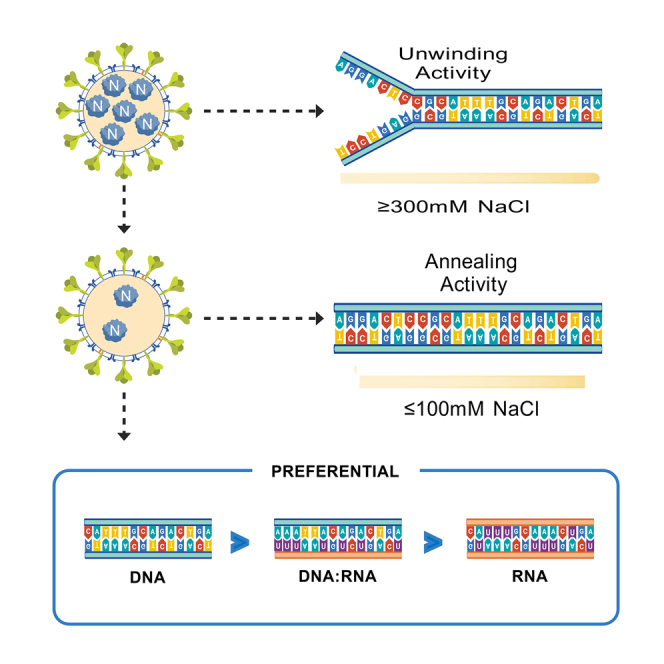

## Introduction

SARS-CoV-2 is the etiological agent of COVID-19, characterized by extreme variability and transmissibility.^1^^,^^2^ SARS-CoV-2, a severe acute respiratory syndrome coronavirus, causes potentially fatal respiratory disease primarily through airborne transmission.^3^ Beyond respiratory symptoms such as acute respiratory distress syndrome and respiratory failure, COVID-19 is now recognized to induce systemic inflammation, which can lead to sepsis, acute cardiac injury, heart failure, and multi-organ dysfunction in high-risk patients. This has resulted in significant damage to global economic development and human life and health.^2^^,^^4^ Since 2020, numerous studies have sought to identify and elucidate the molecular mechanisms of COVID-19 pathogenesis, immune evasion, and disease progression, but these mechanisms remain incompletely understood.^5^^,^^6^ SARS-CoV-2 is a β-coronavirus with a single-stranded positive-sense RNA genome, the largest known among RNA viruses.^7^^,^^8^ It contains four structural proteins—spike (S), envelope (E), membrane (M), and nucleocapsid (N)—along with 16 nonstructural proteins (NSP1-16) essential for replication and transcription.^9^^,^^10^ These proteins share a high degree of sequence similarity with those of SARS-CoV and MERS-CoV, suggesting common pathogenesis. However, SARS-CoV-2 exhibits greater virulence and transmissibility compared to SARS-CoV.^11^ This difference in pathogenicity may arise from interactions between viral proteins and host proteins, which influence viral entry and tissue spread.^12^^,^^13^^,^^14^ Understanding these protein-protein interactions is crucial not only for understanding viral infections but also for the development of antiviral strategies. Among these proteins, the SARA-CoV-2 nucleocapsid protein (hereinafter referred to as CoV-2 N) is one of the most abundant and plays a critical role in the viral life cycle, performing multiple functions during infection.^15^^,^^16^^,^^17^

CoV-2 N is a ∼46 kDa multi-structural domain, highly basic, RNA-binding protein consisting of three main structural domains: an N-terminal structural domain (NTD), which primarily recognizes and binds to DNA/RNA of specific or non-specific sequences, and has been considered a target for inhibitor studies.^18^^,^^19^ A C-terminal structural domain (CTD), which is mainly involved in dimerization/oligomerization and has the potential to interact with other molecules, and a disordered structural domain, the intrinsically disordered region (IDR), which acts as a linker between the NTD and CTD, limiting their interaction.^20^^,^^21^^,^^22^ The NTD of CoV-2 N contains two RNA-binding domains, termed RBD1 and RBD2, which can bind to different dsRNAs, allowing coronaviruses to perform multiple functions in the cytoplasm and facilitating complex biochemical reactions.^23^ CoV-2 N can interact with DDX1, DDX3, DDX5, DDX6, and DDX21 helicases, disrupting mRNA aggregation in an RNA-independent manner,^8^ DDX helicases can act as regulators, modulating host innate immunity and viral amplification both positively and negatively.^24^ For example, DDX1 and the NTD of CoV-2 N can form a DDX1-N heterodimer, which prevents CoV-2 N from binding to genomic RNA (gRNA) and forming oligomers, thereby interfering with SARS-CoV-2 infection and potentially inhibiting viral propagation.^25^ Although DDX1 plays a positive regulatory role in viral resistance, it also serves as a cofactor for viral replication, interacting with CoV-2 N to increase its affinity for ssRNA/dsRNA by 2- to 4-fold, thereby accelerating oligomer complex formation.^26^ Consequently, CoV-2 N can play a major role in viral replication, promoting infection through protein-protein interactions and suppressing the host’s innate immune response.^8^^,^^27^ The high sequence similarity of these proteins with the corresponding sequences of SARS-CoV and MERS-CoV suggests common pathogenic mechanisms. During SARS-CoV replication, the interaction between Nsp13 and DDX5 has been shown to promote coronavirus infection, suggesting that host helicases may act as co-activators to enhance viral genome transcription and viral replication.^28^ The N protein is the central hub of these DDX interactions. SARS-CoV-2 Nsp13(hereinafter referred to as CoV-2 Nsp13), a highly conserved non-structural protein in SARS-CoV-2, plays an essential role in viral replication, homologous recombination, and nucleic acid metabolism.^29^ CoV-2 Nsp13 interacts with host deubiquitylating enzymes (USP13), hijacking the host system to prevent its degradation,^30^ while also leading to the activation of the DNA damage-responsive kinases CHK1 and TANK-binding kinase 1 (TBK1) through the autophagy pathway. This activation inhibits the production of host type I IFNs. Both CoV-2 Nsp13 and CoV-2 N are considered promising targets for antiviral drug development.^31^^,^^32^ CoV-2 N has opened a new pathway in the viral replication cycle by facilitating double-stranded RNA (dsRNA) melting and transferring the nascent strand to transcriptional regulatory sequences located at the 5′ end of each open reading frame. It has also been found to facilitate the template switching of transcriptional regulatory sequences (TRSs) in viral discontinuous RNA transcripts, accelerating the packaging process and viral replication, thus supporting multiple viral functions.^33^^,^^34^^,^^35^

In the functional system of helicases, unwinding and annealing are two biologically antagonistic yet coordinated, unified activities. The unwinding activity relies on ATP hydrolysis to separate double-stranded nucleic acids and release single-stranded templates, whereas the annealing activity promotes the rehybridization of complementary single strands, maintaining the dynamic equilibrium of nucleic acid structures. The balance between these two activities is finely regulated by ATP concentration and the ionic environment—when divalent metal ions (typically Mg^2+^) are present, helicases can hydrolyze ATP to release energy and open double-stranded substrates. Under physiological conditions, this dual activity allows helicases to cooperatively participate in processes such as DNA replication, damage repair, and RNA metabolism. For example, at the replication fork, the helicase unwinds the double strand while its annealing activity prevents excessive exposure to single-stranded regions, thereby avoiding genomic instability.

To comprehensively investigate the multifunctional properties of CoV-2 N in nucleic acid interactions, this study designed a variety of nucleic acid substrates, including blunt, fork, overhang, and G-quadruplex (G4) structures. G4 structures, as key elements of gene regulation, play important roles in viral genome replication, transcription, and translation, and their unique higher-order structures may significantly influence helicase/annealing activities.^36^^,^^37^^,^^38^^,^^39^ Therefore, incorporating G4 substrates helps to reveal the functional specificity of CoV-2 N on complex nucleic acid structures, providing new insights into its roles in viral replication and host interactions. Based on this, we further elucidated the dual regulation of CoV-2 N’s unwinding and annealing activities by protein concentration and ionic strength.

When comparing RNA substrates, it was found that low concentrations of CoV-2 N preferentially favor the annealing of DNA substrates. This suggests that CoV-2 N may anneal nucleic acid substrates through a different mechanism, possibly due to its dual role in viral assembly within the host and providing nucleic acid substrates for CoV-2 Nsp13-mediated deconjugation, thereby evading host immune defenses. Moreover, CoV-2 N exerts its annealing activity on nucleic acid substrates in a manner that is non-directional. Interestingly, *in vitro* experiments revealed that CoV-2 Nsp13 could regulate CoV-2 N deconjugation and annealing, but CoV-2 N had no reciprocal effect on CoV-2 Nsp13 activity. These findings provide new insights into the interaction mechanisms between CoV-2 N and other viral proteins, as well as with host proteins.

## Results

### Fluorescence anisotropy determination of CoV-2 N and CoV-2 Nsp13 binding affinity to nucleic acid substrates

**Figure 1:**
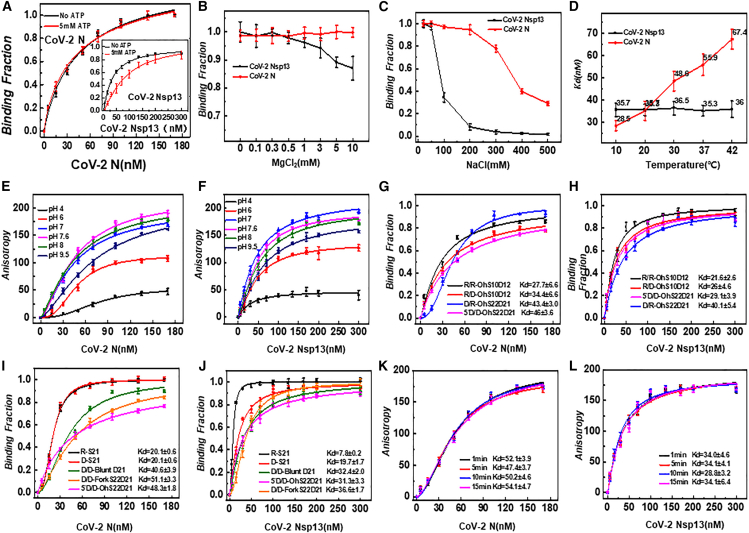
Comparison of the binding activity between CoV-2 N and CoV-2 Nsp13 (A–F) Comparison of the effects of ATP (A), MgCl_2_ (B), NaCl (C), temperature (D), and pH (E, F) on the binding activity of CoV-2 N and CoV-2 Nsp13. (G and H) Comparison of the substrate binding preferences of CoV-2 N and CoV-2 Nsp13 for different structural substrates. (I and J) Comparison of the binding activity of CoV-2 N and CoV-2 Nsp13 on different conformational substrates. (K and L) Effect of incubation time on the binding activity of CoV-2 N and CoV-2 Nsp13. All experiments were conducted under standard conditions. The substrate concentration was 5 nM, with a FAM fluorescent label at the 3′end. Detailed experimental conditions are described in the “Materials and Methods” section. The substrates used in (A–F, K–L) were 5′D/D-OhS22D21. In (A) the composition of the reaction solution for the red curve is: 50 mM NaCl, 25 mM Tris-HCl, pH 7.6.5 mM ATP. The composition of the reaction solution for the black curve is: 50 mM NaCl, 25 mM Tris-HCl, pH 7.6.

Both CoV-2 N and CoV-2 Nsp13 have nucleic acid binding capacities, and the degree of binding directly affects their unwinding efficiency. The unwinding activity is typically influenced by factors such as ATP, salt concentration, Mg^2+^, temperature, pH, and nucleic acid structure. Therefore, we first measured the effects of different conditions on the nucleic acid binding activity of CoV-2 N and CoV-2 Nsp13 using 5′D/D-OhS22D21 in a fluorescence polarization experiment. As motor proteins, helicases hydrolyze ATP in the presence of Mg^2+^ to generate the energy required for duplex unwinding. To avoid the simultaneous occurrence of unwinding during binding assays, Mg^2+^ was excluded, thereby allowing the accurate assessment of binding efficiency independent of unwinding activity. The results showed that the binding activity of CoV-2 N was not significantly affected in the presence of 5 mM ATP, whereas 5 mM ATP had a pronounced inhibitory effect on the binding activity of CoV-2 Nsp13 (Figure 1A). The Mg^2+^ concentration up to 10 mM did not affect the binding ratio of CoV-2 N, whereas the binding activity of CoV-2 Nsp13 was clearly inhibited by Mg^2+^ (Figure 1B). The inhibition of CoV-2 Nsp13 binding activity by 100 mM NaCl was approximately 65%, and CoV-2 Nsp13 was almost completely dissociated from the nucleic acid substrate at NaCl concentrations greater than 200 mM. In contrast, CoV-2 N exhibited strong tolerance to NaCl, with NaCl concentrations below 200 mM having virtually no effect on its binding viability. Only when NaCl exceeded 300 mM did the binding activity of CoV-2 N show a noticeable decrease (Figure 1C). There was no significant change in the binding activity of CoV-2 Nsp13 to the substrate between 10°C and 42°C, whereas CoV-2 N showed higher binding activity at lower temperatures, with the activity decreasing as the temperature increased (Figure 1D). The effect of pH on the binding of CoV-2 N and CoV-2 Nsp13 to substrates exhibited similar properties. Both showed a significant decrease in anisotropy values at pH 6, with almost complete dissociation from the DNA at pH 4, and demonstrated a greater ability to bind to substrates in neutral or slightly alkaline environments (Figures 1E and 1F). The binding of CoV-2 N to different types of nucleic acid substrates revealed that both CoV-2 N and CoV-2 Nsp13 showed a stronger preference for binding to purely conformational RNA substrates and comparatively less affinity for purely conformational DNA substrates (Figures 1G and 1H). Examination of different configurations of nucleic acid substrates revealed that both CoV-2 N and CoV-2 Nsp13 exhibited a pronounced preference for single-stranded nucleic acid substrates, whether RNA or DNA, with no significant difference in binding to other structures (Figures 1G and 1H, 1I, and 1J). As the incubation time was extended from 1 min to 15 min, the affinity of both CoV-2 N and CoV-2 Nsp13 for the substrate remained essentially unchanged, indicating that the maximum binding affinity was achieved within the first minute (Figures 1K and 1L).

### Determination of the specificity and kinetics of CoV-2 N annealing activity on nucleic acid substrates

**Figure 2:**
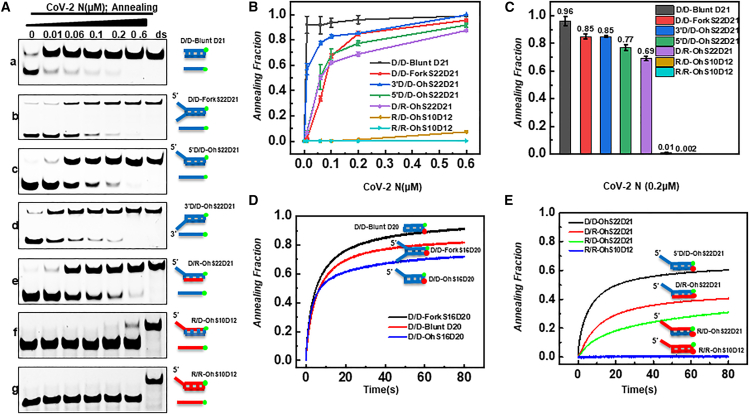
Analysis of the annealing activity of CoV-2 N on different substrates (A) Electrophoretic analysis of the annealing activity of CoV-2 N on different substrates. Panels a–g represent the following substrates in sequence: blunt-ended dsDNA, fork-structured tail strand (22 nt), dsDNA with a 21-bp duplex, dsDNA with a 5′overhang, dsDNA with a 3′overhang, DNA-RNA hybrid with a 5′DNA overhang, RNA-DNA hybrid with a 5′RNA overhang, and dsRNA. Lanes labeled “ss” contain complementary single-stranded nucleic acids, while lanes labeled “ds” contain duplex substrates. All substrates were used at a concentration of 10 nM, with increasing protein concentrations. (B) Densitometric analysis of electrophoretic results for CoV-2 N on different substrates. (C) Bar graph comparing the annealing activity of CoV-2 N (0.2 μM) on different substrates. (D and E) Real-time monitoring of CoV-2 N annealing activity using stopped-flow technology, with a fixed substrate concentration of 4 nM and a protein concentration of 0.6 μM. All experiments were conducted under the conditions described in the “Materials and Methods” section.

In this part of the experiment, we analyzed the annealing activity and kinetic parameters of CoV-2 N on different types and structures of substrates. The results showed that CoV-2 N exhibited strong annealing activity against pure DNA blunt-end (Figure 2Aa), fork (Figure 2Ab), and overhang (Figure 2Ac-d) structures. Among the tested substrates, CoV-2 N exhibited the highest annealing activity against blunt-end structures, with the annealing rate reaching as high as 96% at a protein concentration of 0.2 μM (Figures 2A–2C). However, CoV-2 N showed almost undetectable annealing activity against pure RNA or RNA tail overhang substrates (Figure 2Af, g). Interestingly, the annealing activity of CoV-2 N appeared to be related to the type of overhang tail. When the overhang was composed of DNA, strong annealing activity was observed, regardless of whether the complementary strand was DNA or RNA (Figure 2Ac, e). In contrast, when the overhang tail was RNA, only weak annealing activity was observed, regardless of whether the substrate was homoduplex or heteroduplex (Figure 2Af, g).

To capture the dynamic process and kinetic parameters of CoV-2 N annealing substrates (Table 1), real-time monitoring of the annealing tendency of CoV-2 N on different structural substrates was performed using the stopped-flow technique. We further analyzed the performance of CoV-2 N on different substrates. Under the same reaction conditions, CoV-2 N exhibited high unwinding efficiency on short-tailed or blunt-ended DNA/DNA substrates (Blunt D20, Fork S16D20, Oh S16D20), with amplitudes ≥0.72 (0.82 ± 0.03, 0.91 ± 0.04, and 0.72 ± 0.01, respectively) and rates ≥0.12 s^−1^ (0.12 ± 0.01, 0.15 ± 0.004, and 0.15 ± 0.02, respectively), indicating that SARS-CoV-2 N exhibits robust and relatively architecture-insensitive activity on different DNA configurations. When the 5′single-stranded (ss) tail was extended to 22 nt (5′D/D-OhS22D21), the amplitude and rate decreased to 0.59 ± 0.02 and 0.059 ± 0.002 s^−1^, respectively, suggesting that the elongation of the ss tail significantly reduces the protein’s processivity and unwinding efficiency. We speculate that this phenomenon is related to the reduced translocation efficiency of N protein on longer ss regions, i.e., the slower translocation rate prolongs the time required for a single translocation and subsequent unwinding. This observation is consistent with previous reports showing decreased helicase processivity on long ssDNA.^40^^,^^41^^,^^42^

**Table 1:** Kinetic analysis of CoV-2 N annealing parameters for different substrates using the stopped-flow technique

Substrate Parameters	D/D-Blunt D20	D/D-ForkS16D20	D/D-OhS16D20	5′D/D-OhS22D21	D/R-OhS22D21	R/D-OhS10D12	R/R-OhS10D12
Amplitude	0.82 ± 0.03	0.91 ± 0.04	0.72 ± 0.01	0.59 ± 0.02	0.41 ± 0.02	0.36 ± 0.03	–
Rate (s-1)	0.12 ± 0.01	0.15 ± 0.004	0.15 ± 0.02	0.059 ± 0.002	0.06 ± 0.03	0.03 ± 0.01	–

Moreover, on RNA-containing hybrid substrates (D/R-OhS22D21, R/D-OhS10D12), the unwinding amplitudes of CoV-2 N were 0.41 ± 0.02 and 0.36 ± 0.03, and the rates were 0.06 ± 0.03 and 0.03 ± 0.01 s^−1^, respectively, significantly lower than the corresponding DNA/DNA controls. This suggests that RNA incorporation may alter the physicochemical properties of the substrate (e.g., rigidity, base stacking, and groove geometry), thereby reducing effective enzyme binding and processivity. On fully RNA duplexes (R/R-OhS10D12), no unwinding activity of CoV-2 N was detected, further supporting that the N protein is highly sequence-sensitive and exhibits a strong preference for DNA substrates.

Overall, CoV-2 N exhibits high unwinding activity on DNA substrates and is relatively insensitive to substrate architecture. However, it is highly sensitive to the length of the ss tail and the presence of RNA sequences; both tail elongation and increased RNA content lead to concomitant reductions in the protein’s unwinding amplitude and rate. The molecular dynamics analysis results were generally consistent with those detected by EMSA experiments. From the annealing parameters, it was evident that the blunt-end DNA double-stranded substrate had the highest annealing rate and amplitude, whereas the RNA double-stranded substrate had the lowest values.

### CoV-2 N unwinding activity

This has piqued our interest, especially given recent reports that CoV-2 N interacts with the host’s DDX helicase, suggesting that it may exert continuous effects within the host.^24^^,^^25^^,^^26^ This section aims to explore the potential interaction between CoV-2 N and the host genome through *in vitro* assays, in which we constructed various nucleic acid substrates and structures to compare the unwinding tendencies of CoV-2 N.

**Figure 3:**
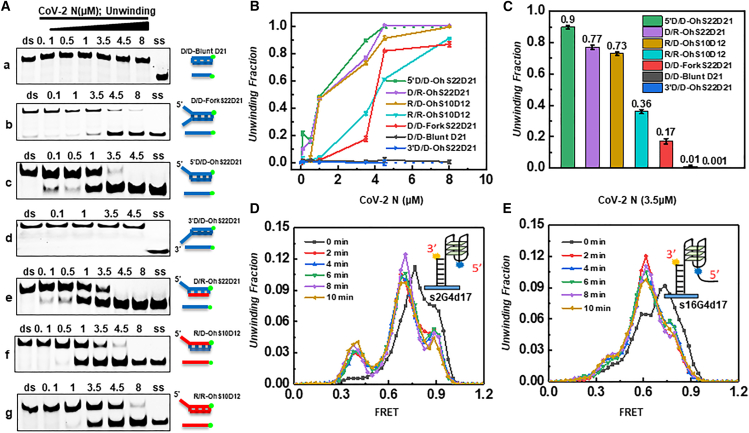
Analysis of the helicase activity of CoV-2 N on different substrates (A) Electrophoretic analysis of the helicase activity of CoV-2 N on different substrates. Panels a–g represent the following substrates in sequence: blunt-ended dsDNA, fork-structured tail strand (22 nt), dsDNA with a 21-bp duplex, dsDNA with a 5′overhang, dsDNA with a 3′overhang, DNA-RNA hybrid with a 5′DNA overhang, RNA-DNA hybrid with a 5′RNA overhang, and dsRNA. Lanes labeled “ds” contain duplex DNA, while lanes labeled “ss” contain complementary single-stranded nucleic acids. All substrates were used at a concentration of 10 nM, with increasing protein concentrations. (B) Densitometric analysis of electrophoretic results for CoV-2 N on different substrates. (C) Bar graph comparing the helicase activity of CoV-2 N (3.5 μM) on different substrates. (D and E) Single-molecule analysis of CoV-2 N on the stability of G4-structured DNA. The black curve represents the control group (0 min, without CoV-2 N), while the colored curves represent FRET values collected at different time points. All experiments were conducted under the conditions described in the “Materials and Methods” section.

To address this question, we constructed various nucleic acid substrates and structures to compare the propensity of CoV-2 N to unwinding. The results showed that CoV-2 N was unable to unwind double-stranded DNA with a blunt-ended structure lacking a tail strand (Fig. 3Aa). When comparing overhang and forked substrates, it was found that CoV-2 N was significantly more efficient at unwinding substrates with a 5′ overhang structure than those with a forked structure. The most efficiently unwound substrate was the pure DNA substrate (Figures 3A–3C).To unambiguously determine whether the observed helicase activity of CoV-2 N arises from residual host-derived non-specific protein contaminants during expression and purification, we engineered a truncated form of the full-length CoV-2 N and purified it using the same protocol. The full-length CoV-2 N is predicted to possess multiple flexible regions, and to date, no complete three-dimensional structure has been reported in the PDB database. To address this, we employed AlphaFold3 to predict the full-length structure and subsequently designed a truncation at the G214 position. The structural prediction of the truncated protein revealed that it encompasses the RNA-binding domain and the dimerization domain of the CoV-2 N. Since dimerization is essential for its role in RNA packaging and viral replication,^22^^,^^43^ this truncated variant is likely deficient in these functions (Figure S1).To further verify the activity of the truncated proteins, we used *in vitro* EMSA to assess the activity of the truncated proteins. The results demonstrated that both truncated constructs lacked helicase activity, supporting the conclusion that the helicase function observed in CoV-2 N is not due to host protein contamination (Figure S2).Surprisingly, the unwinding rate of RNA substrates by CoV-2 N was significantly lower than that of DNA substrates. At a concentration of 3.5 μM, CoV-2 N was able to unwind 90% of the D/D-OhS22D21 substrate, whereas only 36% of the R/R-OhS10D12 substrate was unwound at the same concentration (Fig. 3Ag, 3C). We additionally designed two typical three-layer G4 substrates. CD measurements indicate that substrate sequences can form characteristic G4 wavelengths (data not shown). In samples with CoV-2 N, the peaks of both G4 substrates shifted significantly over time but did not completely transform, suggesting that under the influence of CoV-2 N, the G4 structure was not fully opened, tending instead to transition into an intermediate unstable state (Figures 3D and 3E).

Based on these findings, we speculate that CoV-2 N may affect the host genome in the following ways: (i) the helicase activity of CoV-2 N is more likely to act on host genomic DNA; (ii) CoV-2 N may target the host genome depending on nucleic acid type and structure; and (iii) CoV-2 N may interact with host helicases through host genome mediation.

### Regulatory mechanisms of CoV-2 N helicase and annealing activity

**Figure 4:**
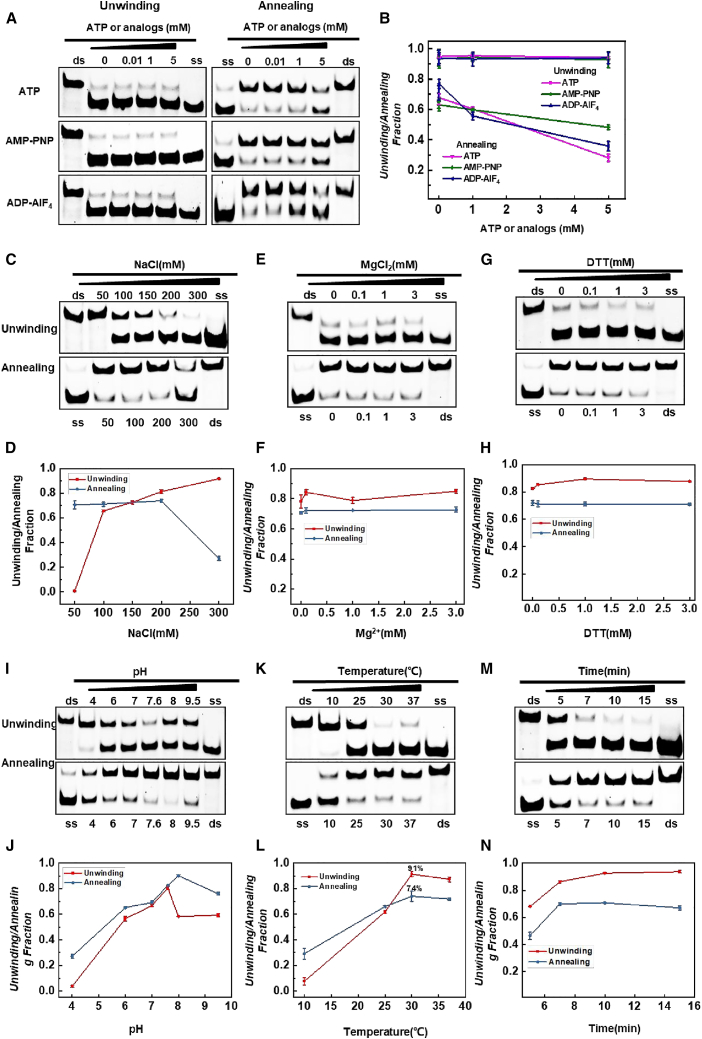
Exploration and comparison of the optimal conditions for CoV-2 N helicase and annealing activities (A) Effects of ATP, AMP-PNP, and ADP-AlF_4_ on the helicase and annealing activities of CoV-2 N. (B–M) Densitometric analysis of (A). (C, E, G, I, K, M) Effects of NaCl (C), MgCl_2_ (E), DTT (G), pH (I), temperature (K), and incubation time (M) on the helicase and annealing activities of CoV-2 N. (D, F, H, J, L, N). Densitometric comparisons corresponding to (C), (E), (G), (I), (K), and (M), respectively. Lanes labeled “ds” represent the 5′D/D-OhS22D21 duplex control, while lanes labeled “ss” represent the FAM-labeled single-stranded control (3′-FAM-S43). The substrate concentration was 10 nM. The concentration of CoV-2 N was 3.5 μM in helicase buffer and 0.2 μM in annealing buffer. All experiments were conducted under the conditions described in the “Materials and Methods” section.

CoV-2 N is not a typical helicase but possesses unwinding and annealing activities. What mechanisms does CoV-2 N use to regulate these two opposing kinetic activities? We compared the efficiency of both activities under different reaction conditions, such as ATP, ATP analogs, NaCl concentration, Mg^2+^, DTT, pH, temperature, and reaction time. It was found that a gradual increase in ATP and ATP analog concentrations had almost no significant effect on the unwinding activity of CoV-2 N,^44^ while the annealing activity was clearly inhibited with increasing concentrations of ATP and analogs (Figures 4A and 4B). CoV-2 N demonstrated better unwinding activity in a 200–300 mM NaCl environment, while its annealing activity was enhanced in a 50–200 mM NaCl environment (Figures 4C and 4D). As the concentrations of Mg^2+^ and DTT increased, there was no significant difference between the unwinding and annealing activities of CoV-2 N, indicating that these activities do not rely on Mg^2+^ and DTT participation (Figures 4E–4H). CoV-2 N exhibited the best enzyme activity at pH 7.6 (Figures 4I and 4J). Both annealing and unwinding activities performed optimally above 30°C, achieving 91% of the maximum effect for unwinding and 74% for annealing (Figures 4K and 4L). The unwinding and annealing activities of CoV-2 N occurred very rapidly, with similar experimental effects observed after 10 min of incubation (Figures 4M and 4N). Therefore, the regulation of unwinding and annealing activities by CoV-2 N differs from that of typical helicases, whose activities are usually governed by ATP and Mg^2+^ concentrations. The results indicate that ATP, MgCl_2_, and DTT show similar trends in unwinding and annealing assays, with annealing activity favoring lower temperatures and unwinding activity being inhibited at the same temperature (25°C). In contrast, the NaCl experiments revealed opposite results, as CoV-2 N showed optimal unwinding activity at 300 mM NaCl while demonstrating the significant inhibition of annealing activity at this concentration. These results suggest that the regulation of unwinding and annealing activities by CoV-2 N may be related to the ionic concentration in the environment.

### The ion concentration and CoV-2 N concentration jointly regulate helicase and annealing activities

**Figure 5:**
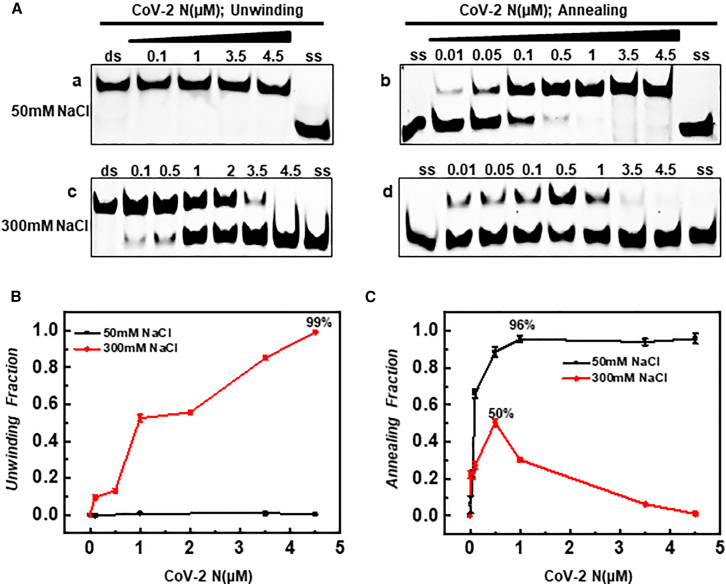
Effects of NaCl on the helicase and annealing activities of CoV-2 N (A) Electrophoretic analysis compares the helicase (a, c) and annealing (b, d) activities of CoV-2 N in buffer conditions containing 50 mM and 300 mM NaCl. The substrate concentration was 10 nM. Lanes labeled “ds” represent the 5′D/D-OhS22D21 duplex control, while lanes labeled “ss” represent the FAM-labeled single-stranded control (3′-FAM-S43). (B and C) Densitometric comparisons corresponding to (A-a, A-c) and (A-b, A-d), respectively. All experiments were conducted under the conditions described in the “Materials and Methods” section.

Although our previous experiments indicated that unwinding and annealing activities are closely related to NaCl concentrations and identified the optimal ranges for both, we also observed that at 200 mM NaCl that both activities could open double-stranded substrates and anneal single-stranded substrates simultaneously (Figure 4C). So, is it possible for these two functions to be exerted simultaneously and also converted? Our previous results demonstrated that under high-salt conditions (300 mM NaCl), the activity profile of CoV-2 N varied in a concentration-dependent manner.^44^ At concentrations below 0.5 μM, annealing activity increased progressively, whereas at concentrations above this threshold, annealing activity was significantly reduced, coinciding with a shift toward enhanced unwinding activity (Figures 5Ad and S3). To further verify this hypothesis, we simultaneously compared two reaction environments: 50 mM NaCl (low salt) and 300 mM NaCl (high salt). The results showed that CoV-2 N had no unwinding activity under low salt conditions but exhibited extremely strong annealing activity, reaching 96% at a concentration of 1 μM (Fig. 5Aa-b). From the plots in (Fig. 5Ac), we can see that the unwinding activity of CoV-2 N under high salt conditions reached 99% at 4.5 μM (Figure 5B). Thus, we can conclude that the unwinding and annealing activities of CoV-2 N are regulated in a dual manner by both protein concentration and ionic strength. We hypothesize that within the SARS-CoV-2 virus, CoV-2 N predominantly exhibits annealing activity. Once the ionic concentration in the environment reaches a certain level, it inhibits the unwinding activity of the viral gene-encoded CoV-2 Nsp13 helicase. In high salt environments, CoV-2 N may enhance its unwinding activity by increasing its concentration, thereby assisting the viral CoV-2 Nsp13 helicase in completing the replication of the viral genome. This could represent another compensatory pathway for viral replication.

CoV-2 Nsp13, as a typical helicase, exerts very strong unwinding activity at a concentration of 50 mM NaCl, whereas CoV-2 N demonstrates strong annealing activity under the same conditions. However, when both coexist in the virus, it remains unclear whether CoV-2 N inhibits the helicase activity of CoV-2 Nsp13 or if, in a high-salt environment, they act synergistically to facilitate viral genome replication. To investigate this, we continued using the overhang structure commonly observed during replication (5′D/D-OhS22D21), and under optimal conditions for CoV-2 Nsp13 and CoV-2 N, respectively, we explored their interactions.

**Figure 6:**
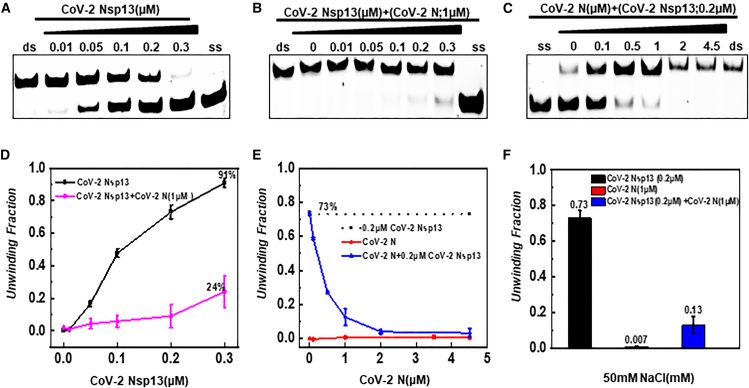
Effect of 50 mM NaCl on the helicase activity of CoV-2 N and its interaction with CoV-2 Nsp13 (A) Helicase activity of CoV-2 Nsp13 alone. (B) Effect of increasing CoV-2 Nsp13 concentrations on substrate unwinding while maintaining a fixed concentration of CoV-2 N (1 μM). Control groups were excluded, and CoV-2 Nsp13 was incrementally added and mixed. (C) Effect of increasing CoV-2 N concentrations on substrate unwinding while maintaining a fixed concentration of CoV-2 Nsp13 (0.2 μM). Control groups were excluded, and CoV-2 N was incrementally added and mixed. (D–F) Densitometric comparisons of (A), (B), and (C), respectively. The helicase activity of CoV-2 Nsp13 (black line), the mixed protein condition (magenta and blue line), and CoV-2 N (red line) are shown. The substrate concentration (5′D/D-OhS22D21) was 10 nM. Lanes labeled “ds” represent the dsDNA duplex control, while lanes labeled “ss” represent the FAM-labeled single-stranded control (3′-FAM-S43). All experiments were conducted under the conditions described in the “Materials and Methods” section.

First, under the optimal helicase buffer for CoV-2 Nsp13 (low-salt: 50 mM NaCl, 25 mM Tris-HCl, pH 7.6, 1.5 mM Mg^2+^, 1 mM DTT), when the CoV-2 Nsp13 concentration reached 0.3 μM, the double-stranded substrate was almost completely unwound. However, when a fixed concentration of CoV-2 N (1 μM) was added to each sample under the same reaction conditions, the helicase activity of CoV-2 Nsp13 was almost completely inhibited (Figures 6A and 6B). To further validate this conclusion, a fixed concentration of CoV-2 Nsp13 (0.2 μM) was included in lanes 2–7 of Figure 6C. In the absence of additional CoV-2 N, the unwinding fraction reached 73% (lane 2). As the concentration of CoV-2 N was gradually increased in lanes 3–7, the unwinding activity of CoV-2 Nsp13 gradually decreased, and at 2 μM CoV-2 N, the unwinding activity of CoV-2 Nsp13 was almost completely inhibited.

**Figure 7:**
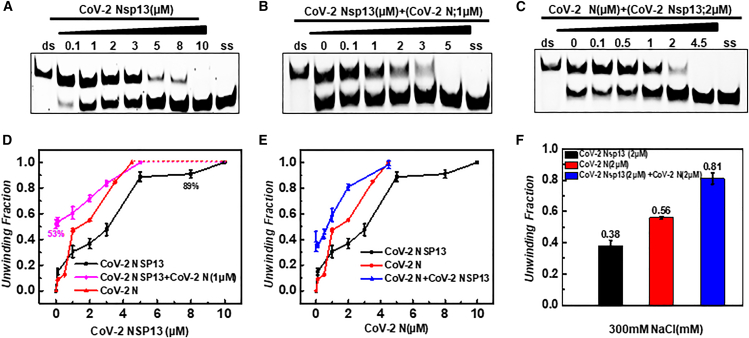
Effect of 300 mM NaCl on the helicase activity of CoV-2 N and its interaction with CoV-2 Nsp13 (A) Helicase activity of CoV-2 Nsp13 alone. (B) Effect of increasing CoV-2 Nsp13 concentrations on substrate unwinding while maintaining a fixed concentration of CoV-2 N (1 μM). Control groups were excluded, and CoV-2 Nsp13 was incrementally added and mixed. (C) Effect of increasing CoV-2 N concentrations on substrate unwinding while maintaining a fixed concentration of CoV-2 Nsp13 (0.2 μM). Control groups were excluded, and CoV-2 N was incrementally added and mixed. (D–F) Densitometric comparisons of (A), (B), and (C), respectively. The substrate concentration (5′D/D-OhS22D21) was 10 nM. Lanes labeled “ds” represent the dsDNA duplex control, while lanes labeled “ss” represent the FAM-labeled single-stranded control (3′-FAM-S43). All experiments were conducted under the conditions described in the “Materials and Methods” section.

Conversely, under the high-salt conditions optimal for CoV-2 N (300 mM NaCl, 25 mM Tris-HCl pH 7.6, 1.5 mM Mg^2+^, 1 mM DTT), although we observed the complete unwinding of the substrate, a much higher concentration of CoV-2 Nsp13 was required—approximately 30 times higher than under low-salt conditions. This indicates that CoV-2 Nsp13’s helicase efficiency is significantly reduced in high-salt environments (Figures 6A, 7A). When a fixed concentration of CoV-2 N (1 μM) was added to each sample, the concentration of CoV-2 Nsp13 required to achieve the same degree of unwinding was significantly lower (Figures 7A and 7B). In samples containing a fixed concentration of CoV-2 Nsp13, a similar additive effect was observed (Figure 7C).To more directly observe the interaction between the two under different conditions, we used line and bar graphs to compare the degree of unwinding when each acted on the substrate individually versus when both acted simultaneously. The results showed that in a low-salt environment, CoV-2 N inhibited CoV-2 Nsp13’s unwinding of the substrate (Figures 6D–6F), while in a high-salt environment (under stress), CoV-2 N and CoV-2 Nsp13 may exhibit additive effects, jointly promoting substrate unwinding (Figures 7D–7F).

### Detection of ATPase activity when CoV-2 Nsp13 and CoV-2 N interact with each other

**Figure 8:**
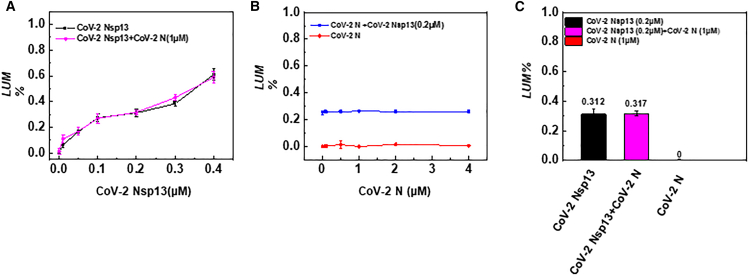
Analysis of ATPase activity in the presence of CoV-2 Nsp13, CoV-2 N alone, and both proteins together (A) Analysis of ATP consumption rate with a fixed CoV-2 N concentration (1 μM) and increasing CoV-2 Nsp13 concentrations. (B) Analysis of ATP consumption rate with a fixed CoV-2 Nsp13 concentration (0.2 μM) and increasing CoV-2 N concentrations. (C) Comparative analysis of ATP consumption when CoV-2 Nsp13 (0.2 μM) and CoV-2 N (1 μM) are present alone or together. For (A–B), the y axis represents ATP consumption percentage (LUM) in the experimental group, while the x axis represents protein concentration. The substrate concentration (5′D/D-OhS22D21) was fixed at 10 nM. All experiments were conducted under the conditions described in the “Materials and Methods” section.

In the previous experiment, we observed that CoV-2 N inhibited the helicase activity of CoV-2 Nsp13 under low-salt conditions. One possible mechanism is that CoV-2 N suppresses the ATPase activity of CoV-2 Nsp13, thereby depriving it of the energy required for helicase activity. Alternatively, CoV-2 N may not directly interact with CoV-2 Nsp13 but instead utilize its strong annealing activity to re-anneal the double-stranded substrate that CoV-2 Nsp13 has unwound. To explore these possibilities, we used an ATP consumption assay to measure ATP utilization in the reaction. The results showed that ATP utilization increased linearly as the concentration of CoV-2 Nsp13 increased (Figure 8A, black line). However, when a fixed concentration of CoV-2 N (1 μM) was added to the same reaction, no significant change in ATP utilization was observed (Figure 8A, magenta line), suggesting that the presence of CoV-2 N does not inhibit the ATPase activity of CoV-2 Nsp13.In a separate control experiment, we varied the concentration of CoV-2 N and found no significant increase in ATP utilization (Figure 8B, red line). Although it has been reported that CoV-2 N contains ATP-binding sites,^45^ our results indicate that CoV-2 N alone does not exhibit ATP hydrolysis activity, consistent with previous findings that CoV-2 N can unwind substrates in the absence of ATP (Figure 4A). Similarly, when CoV-2 Nsp13 concentration was fixed at 0.2 μM, ATP utilization remained stable at around 30% and did not decrease as CoV-2 N concentration increased (Figure 8B, blue line). Additionally, CoV-2 Nsp13 at 0.2 μM showed little difference in ATPase activity compared to the mixed protein samples, and CoV-2 N alone had minimal ATP utilization ability (Figure 8C). These results suggest that CoV-2 N and CoV-2 Nsp13 do not interact directly through spatial structural interaction.

## Discussion

The COVID-19 pandemic, caused by the SARS-CoV-2 virus, has inflicted significant social harm on a global scale.^46^ A deeper understanding of the replication mechanisms of SARS-CoV-2 can provide valuable insights for future antiviral treatments. The virus-encoded CoV-2 N is one of the structural proteins of SARS-CoV-2, playing a critical regulatory role in viral replication, assembly, immune evasion, and pathogenesis,^47^^,^^48^ and is thus considered an ideal antiviral drug target.^49^^,^^50^^,^^51^ Both CoV-2 N and CoV-2 Nsp13 are multifunctional proteins, and studies have revealed that both possess helicase and annealing activities, though the environments in which they function differ markedly. Whether this unique characteristic of CoV-2 N, which distinguishes it from typical helicases, suggests a special physiological significance within the virus or host cells remains an intriguing question.

How does CoV-2 N regulate its helicase and annealing activities without utilizing ATP as an energy source? Does a functional interaction exist between CoV-2 N and CoV-2 Nsp13 that regulates viral replication and assembly? To address these unanswered questions, we systematically compared CoV-2 N and CoV-2 Nsp13: (1) CoV-2 N displays a certain preference for the type and structure of nucleic acid substrates it acts upon; (2) Specific pH levels and temperatures significantly enhance CoV-2 N’s enzymatic activity; (3)The regulation of CoV-2 N’s helicase and annealing activities is influenced by ionic concentrations and CoV-2 N levels in the environment; (4) Both proteins exhibit additive effects when presented in the same reaction system. Based on these findings, we have gained a deeper understanding of the enzymatic properties of CoV-2 N and its interaction mechanism with CoV-2 Nsp13, providing an attractive target for future antiviral drug development.

This study shows that the binding activity of CoV-2 N to nucleic acid substrates is significantly more tolerant to Na^+^ and Mg^2+^ ion concentrations than CoV-2 Nsp13 (Figure 1B and 1C). The ion concentration and environmental conditions of host cells may vary greatly, and the high tolerance of CoV-2 N to these ionic fluctuations ensures that the protection and packaging of viral RNA are not significantly affected across a wide range of conditions, whereas the sensitivity of CoV-2 Nsp13 to changes in the ionic environment may limit its functional versatility.^52^

It has been reported that CoV-2 N forms aggregate with viral RNA through liquid-liquid phase separation (LLPS) at a specific temperature. These aggregates promote the packaging of the viral genome, the assembly of viral particles, and the nucleic acid binding activity. Under high temperature conditions, the morphology and stability of the aggregates may be affected.^23^^,^^48^^,^^53^ Combined with the results of this study, compared with the high stability of CoV-2 Nsp13 at temperature changes, CoV-2 N is extremely sensitive to high temperatures and prefers low temperature environments (Figure 1D). This phenomenon may be due to high temperatures changing the conformation of CoV-2 N, which in turn triggers its dissociation and ultimately leads to a decrease in binding activity. This study found that CoV-2 N and CoV-2 Nsp13 both showed high binding activity under weakly alkaline physiological conditions (Figures 1E and 1F). This environment may help the formation of CoV-2 N aggregates,^54^^,^^55^^,^^56^ thereby increasing its DNA binding activity and promoting viral replication and spread in host cells.

Currently, the proteins reported to have annealing activity in the SARS-CoV include N protein, NSP7, and NSP8.^44^^,^^57^^,^^58^ The substrate recognition mechanism of N protein has not yet been clarified. Experimental results show that CoV-2 N exhibits high annealing and unwinding activity for substrates of DNA overhang tail chains, and there is no polarity difference in the annealing process (Figures 2A, 3A). The substrate activity for RNA overhang tail chains is weaker, which may be to avoid its annealing activity, leading to the formation of RNA secondary structure in its own genome. The substrate preference of CoV-2 N for DNA tail chains suggests the complex multifunctionality of CoV-2 N in the viral life cycle and the diversity of its interactions with the host.

The G4 structure is believed to participate in and affect viral replication, regulate virulence, and regulate other key steps in the viral life cycle.^59^^,^^60^ The non-structural protein CoV-2 Nsp13 encoded by the SARS-CoV-2 gene contains a domain that can specifically bind to G4 and plays a key role in viral replication and transcription mechanisms.^61^^,^^62^ This study shows that CoV-2 N does not completely open the G4 structure, but it can be clearly observed that the G4 structure becomes unstable (Figures 3D and 3E). The loss of the steady-state G4 structure is more conducive to the process of CoV-2 Nsp13 opening the G4 structure, thereby ensuring the effective packaging and integration of viral RNA and improving replication efficiency.

CoV-2 N exhibits unique enzymatic characteristics that deviate from typical helicases, allowing the virus to adapt to diverse host environments, which is crucial given the varied immune responses of different hosts.^52^ Notably, when ATP levels are insufficient for CoV-2 Nsp13 to unwind the viral genome, CoV-2 N can temporarily compensate without requiring energy, thus maintaining replication efficiency. Furthermore, changes in host cell ion concentrations directly affect CoV-2 Nsp13’s helicase efficiency, with CoV-2 N complementing optimal ion concentrations during unwinding (as depicted in Figure 4A). Experimental results indicate that the regulation of CoV-2 N’s unwinding and annealing activities is closely tied to ion concentration and protein levels.^35^^,^^53^^,^^58^ In low-salt environments, CoV-2 N’s positively charged surface forms strong electrostatic interactions with the negatively charged nucleic acids, stabilizing the double-stranded structure and promoting annealing. Under high-salt conditions, CoV-2 N tends to form multimers, which may be critical for helicase activity, while lower protein concentrations result in monomers or oligomers that sustain annealing. This ion-dependent regulatory mechanism optimizes the virus’s replication and assembly across various cellular environments, enhancing its functional adaptability and sophisticated regulation strategies.

Notably, the concentration threshold (approximately 0.5 μM) at which the CoV-2 N switches from annealing to unwinding activity was determined under specific *in vitro* conditions (300 mM NaCl and a defined DNA substrate). Such transitions are generally influenced by the substrate environment and stoichiometry, rather than by a fixed concentration. Nevertheless, this concentration-dependent functional switch is likely to be physiologically relevant. In infected cells, CoV-2 N is extremely abundant, reaching approximately 10^8^ copies per cell (around 1 mM),^63^ thereby providing a molecular basis for concentration-dependent regulation. The switch may reflect changes in the oligomeric state: high concentrations favor higher-order multimers and unwinding activity, whereas monomers or small oligomers at lower concentrations promote annealing. Oligomerization is further modulated by factors such as ionic strength, pH, and RNA binding.^23^^,^^53^ Moreover, the nuclear localization of CoV-2 N, particularly during the S phase when DNA replication is active,^64^ suggests that its unwinding activity may contribute to the viral hijacking of host-cell processes.

The results demonstrate that CoV-2 N and CoV-2 Nsp13 exhibit mutual effects in both high-salt and low-salt environments (Figures 6, 7). However, the affinity of both proteins for the same nucleic acid substrates does not show significant differences, suggesting that competitive inhibition between the two on the substrate is unlikely (Figures 1E–1L). CoV-2 N also does not affect the ATPase activity of CoV-2 Nsp13, indicating that CoV-2 N does not impair the helicase activity of CoV-2 Nsp13 by altering its core domain (Figure 8). We hypothesize that, in a low-salt environment, CoV-2 N functions similarly to the single-stranded DNA-binding protein (SSB) in prokaryotic replication, where CoV-2 Nsp13 remains at the replication fork to unwind the double strand, while CoV-2 N binds to the single-stranded region ahead of the fork, thereby accelerating the viral replication process. The inhibitory effect observed *in vitro* is likely due to its strong annealing activity, which continuously reanneals the unwound strands back into a double-stranded form. In a high-salt environment, where the helicase activity of CoV-2 Nsp13 is weaker (Figure 7), the virus likely modulates the concentration of CoV-2 N to assist CoV-2 Nsp13 in ensuring the integrity and replication efficiency of the viral RNA.^19^^,^^65^^,^^66^

CoV-2 N in coronaviruses exhibits extremely high homology, suggesting significant functional similarity and importance.^35^ This high degree of conservation makes CoV-2 N an important target for antiviral drug and vaccine development. This study systematically reveals the enzymatic properties of CoV-2 N and CoV-2 Nsp13 under different conditions and identifies potential regulatory mechanisms of the two activities of CoV-2 N. These findings provide insight into the adaptive strategies of coronaviruses in various physiological environments and contribute to the understanding of their survival mechanisms in hosts. Moreover, this work offers a valuable theoretical foundation for future antiviral research.

The primary function of the N protein is to bind and package the viral RNA genome, forming a ribonucleoprotein complex (RNP) and protecting the RNA from degradation. This function is highly conserved in SARS-CoV-2 and other coronaviruses.^35^^,^^53^^,^^67^^,^^68^ Meanwhile, CoV-2 Nsp13, as a helicase, is responsible for unwinding RNA or DNA secondary structures to facilitate replication and transcription. Is there a functional correlation between the two proteins? To investigate this, we designed both forward and reverse experiments under low-salt conditions. In the forward experiment, at a saturating concentration of 1 μM, where CoV-2 N exhibits the strongest annealing activity, the helicase activity of CoV-2 Nsp13 was completely inhibited. In the reverse experiment, at a concentration of 0.2 μM, which corresponds to the highest helicase activity of CoV-2 Nsp13, its helicase activity was progressively inhibited with a gradual addition of CoV-2 N (Figure 6A–6D).

This phenomenon indicates that the annealing activity of CoV-2 N competitively inhibits the helicase activity of CoV-2 Nsp13. We propose three possible inhibitory mechanisms: 1) CoV-2 N competes with CoV-2 Nsp13 for binding to the same DNA site. The high affinity of CoV-2 N for DNA prevents CoV-2 Nsp13 from effectively binding to DNA, thereby inhibiting its helicase activity. However, spectrophotometric analysis revealed no significant differences in their affinities for double-stranded substrates, ruling out the possibility that competitive inhibition is driven by differences in substrate affinity. 2) CoV-2 N and CoV-2 Nsp13 bind to different DNA sites, with CoV-2 Nsp13 targeting the double-stranded region for unwinding, while CoV-2 N functions on all single-stranded regions within the system, exhibiting annealing activity. In this case, CoV-2 N acts similarly to single-stranded binding (SSB) proteins, continuously reannealing single-stranded DNA into double strands. Since the annealing rate of CoV-2 N is faster than the unwinding rate of CoV-2 Nsp13, the overall effect is the inhibition of CoV-2 Nsp13’s helicase activity. 3) CoV-2 N interacts directly with CoV-2 Nsp13, forming a CoV-2 N-Nsp13 complex that alters CoV-2 Nsp13’s functional activity. This direct protein-protein interaction may induce conformational changes in CoV-2 Nsp13, leading to the inhibition of its helicase activity.

### Limitations of the study

It should be emphasized that this study primarily investigates the interaction and enzymatic properties of CoV-2 N and CoV-2 Nsp13 in an *in vitro* system. While these experiments provide important insights into their molecular mechanisms, certain limitations remain. First, the *in vitro* environment cannot fully recapitulate the complex physiological conditions of the host cells, such as macromolecular crowding, organelle compartmentalization, and regulation by multiple co-factors. Second, although the protein concentrations and ionic conditions used in our experiments were optimized, they may still differ from the actual physiological conditions during viral infection. Furthermore, the spatiotemporal distribution and regulatory network of these two proteins during the viral replication cycle are difficult to fully reconstruct *in vitro*. Therefore, future studies employing cellular or animal models will be required to further validate the physiological relevance of these interactions, enabling a more comprehensive assessment of their biological functions and therapeutic potential.

## Resource availability

### Lead contact

Requests for further information should be directed to and will be fulfilled by the lead contact Bozhang, E-mail: bozhangzmu@163.com.

### Materials availability

This study did not generate unique reagents. All the materials of this study are available from the lead contact without restriction upon request.

### Data and code availability

• Data: All data reported in this article will be shared by the lead contact upon request.
• Code: No original code was generated for this study.
• Additional information: Additional information on the data reported in this article and their analysis is available from the lead contact on request.

## Acknowledgments

10.13039/501100004001Guizhou Provincial Science and Technology Plan Foundation (QKHJC-ZK [2023]520), (QKHJC-[2024] youth325);The 10.13039/100014718National Natural Science Foundation of China (32460227), (32160826); The Internal Postdoctoral Training Funding Program of 10.13039/501100019350ZunYi Medical University (ZYBSH[2024]025), (ZYBSH[2024]007).

## Author contributions

B.Z., Y.L., Y.X., and Y-X.D.: conceptualization, supervision, and writing – review and editing. P.Z., Z-L.L., C-H.Y., Y.Z., L.G, H-Y.W., C.M., S-Z.W., and C-L.R.: experimental operation, data collection, and analysis. Y-X.D., J-D. L., and Y.L.: funding acquisition. All the authors have read and agreed to the published version of the article.

## Declaration of interests

The authors declare no conflict of interest.

## STAR★Methods

### Key resources table

**Table:** 

REAGENT or RESOURCE	SOURCE	IDENTIFIER
**Bacterial and virus strains**		

*E. coli BL21(DE3)*	Merck Millipore	NA

**Chemicals, peptides, and recombinant proteins**		

Ampicillin	Sangon Biotech (Shanghai) Co., Ltd.	Cat# D613022
Isopropyl-β-D-thiogalactose pyranoside	Sangon Biotech (Shanghai) Co., Ltd.	Cat# D265334
ATP	Sigma-Aldrich	Cat# A2383
DTT	Solarbio	Cat# D8010
Polyacrylamide	Sangon Biotech (Shanghai) Co., Ltd.	Cat# A100283
Nacl	Sigma-Aldrich	Cat# S9888
Tris-HCl	Solarbio	Cat# T8260
MgCl_2_	Solarbio	Cat# M8150

**Critical commercial assays**		

ATPase Assay Kit	Shanghai Beyotime Biotechnology Co., Ltd.	S0026

**Oligonucleotides**		

5′-CGATGTTTTATTTACATTGTA-3′-F	Sangon Biotech (Shanghai) Co., Ltd.	D-S21
5′-UACAAUGUAAAUAAAACAUCG -3′-F	Sangon Biotech (Shanghai) Co., Ltd.	R-S21
5′-CGATGTTTTATTTACATTGTA-3′-F 3′-GCTACAAAATAAATGTAACAT-5′	Sangon Biotech (Shanghai) Co., Ltd.	D/D-Blunt D21
5′-CTGTAGGAATGTGAAATAAAAACGATG TTTTATTTACATTGTA-3' -F 3' – A(22)GCTACAAAATAAATGTAACAT-5′	Sangon Biotech (Shanghai) Co., Ltd.	D/D-ForkS22D21
5′-CTGTAGGAATGTGAAATAAAAACGATG TTTTATTTACATTGTA-3′-F 3′-GCTACAAAATAAATGTAACAT -5′	Sangon Biotech (Shanghai) Co., Ltd.	5′D/D-OhS22D21
5′-CGATGTTTTATTTACATTGTA-3′-F 3′-T(22)GCTACAAAATAAATGTAACAT -5′	Sangon Biotech (Shanghai) Co., Ltd.	3′D/D-OhS22D21
5′- CTGTAGGAATGTGAAATAAAAACGATG TTTTATTTACATTGTA-3′-F 3′- GCUACAAAAUAAAUGUAACAU-5′	Sangon Biotech (Shanghai) Co., Ltd.	D/R-OhS22D21
5' - UUUUUUUUUUCUCUGCUCGACG-3' -F 3' - GAGACGAGCUGC-5′	Sangon Biotech (Shanghai) Co., Ltd.	R/R-OhS10D12
5′-TTATGTCATTCCGGCAGATG -3′-F 3′-AATACAGTAAGGCCGTCTAC -5′-HF	Sangon Biotech (Shanghai) Co., Ltd.	D/D-Blunt D20
5′-ATCCTATCGAAGAATGTTATGTCATTCC GGCAGATG-3′-F 3′-AATACAGTAAGGCCGTCTAC-5′-HF	Sangon Biotech (Shanghai) Co., Ltd.	D/D-OhS16D20
5' - ATCCTATCGAAGAATGTTATGTCATTCC GGCAGATG-3' -F 3' - T(16)AATACAGTAAGGCCGTCTAC-5′-HF	Sangon Biotech (Shanghai) Co., Ltd.	D/D-ForkS16D20
5′-CTGTAGGAATGTGAAATAAAAACGATGT TTTATTTACATTGTA-3′-F 3′-GCTACAAAATAAATGTAACAT -5′-HF	Sangon Biotech (Shanghai) Co., Ltd.	5′D/D-OhS22D21
5′-CTGTAGGAATGTGAAATAAAAACGATGT TTTATTTACATTGTA-3′-F 3′- GCUACAAAAUAAAUGUAACAU-5′-HF	Sangon Biotech (Shanghai) Co., Ltd.	D/R-OhS22D21
5′-CUGUAGGAAUGUGAAAUAAAAACGAUG UUUUAUUUACAUUGUA -3' -F 3' -GCTACAAAATAAATGTAACAT -5′-HF	Sangon Biotech (Shanghai) Co., Ltd.	R/D-OhS22D21
5' - UUUUUUUUUUCUCUGCUCGACG-3' -F 3' - GAGACGAGCUGC-5′-HF	Sangon Biotech (Shanghai) Co., Ltd.	R/R-OhS10D12
5′-TTTTTTTTTTTTTTTTTTGGGTTAGGGTTAG GGTTAGGGTTGAGGACAC GTGCATTCC-3′ 3' -CTCCTGTGCACGTAAGG-Biotin-5′	Sangon Biotech (Shanghai) Co., Ltd.	S16G4d17
5' -TTGGGTTAGGGTTAGGGTTAGGGTTGAG GACACGTGCATTCC-3′ 3' -CTCCTGTGCACGTAAGG-Biotin-5′	Sangon Biotech (Shanghai) Co., Ltd.	S2G4d17

**Recombinant DNA**		

pET-28a-2019-nCoV-N	Anhui General Biosciences Co., Ltd.	GenBank accession number: NC_045512.2
pSmart-I-CoV-2 Nsp13	Anhui General Biosciences Co., Ltd.	GenBank accession number: OM019196.1

**Software and algorithms**		

Image Lab	Bio-rad	NA
Origin 2024	OriginLab Corporation	NA
SPSS Statistics 29	IBM	NA

**Other**		

Ni-NTA	GE Healthcare	His Trap FF

### Experimental model and study participant details

The pET28a-SARS-CoV-2 N and pSmart-I-CoV-2 Nsp13 plasmids were transformed into *E. coli BL21 (DE3)* for protein expression via heat shock. The cells were cultured in LB medium supplemented with 50 μg/mL kanamycin, and protein expression was induced with 0.3 mM and 0.6 mM IPTG, respectively, followed by overnight incubation at 18°C. After fermentation, the cells were harvested by centrifugation.

### Method details

#### Reagents and buffers

All chemicals were of reagent grade. Buffers were prepared using high-quality deionized water from the Milli-Q Ultrapure Water Purification System (Millipore, Burlington, MA, USA), with a resistivity greater than 18.2 MΩ cm, and further filtered with 2-μm filters before use.

Buffer A: 50 mM NaCl, 25 mM Tris-HCl, pH 7.6.

Buffer B: 100 mM NaCl, 25 mM Tris-HCl, pH 7.6.

Buffer C: 300 mM NaCl, 25 mM Tris-HCl, pH 7.6.

Buffer D: 30 mM NaCl, 25 mM Tris-HCl, pH 7.6, 5 mM MgCl_2_, 1 mM DTT.

Termination buffer:50 mM EDTA, 2% SDS, 30% glycerol, 0.01% bromophenol blue.

#### Recombinant plasmids

(1) The plasmid pET-28a-2019-nCoV-N, kindly provided by the Guangdong Institute of Microbiology, contained the CoV-2 N coding sequence (GenBank accession number: NC_045512.2) with a 6-his tag cloned downstream of the AUG promoter. (2) The pSmart-I-CoV-2 Nsp13, which contained the CoV-2 Nsp13 coding sequence (https://www.ncbi.nlm.nih.gov/nuccore/OM019196.1) and an N-terminal SUMO fusion tag, cloned downstream of the AUG promoter in the expression vector, was synthesized by General Biosystems (Anhui, China) Co., Ltd. (3) The pET28a-CoV-2 N and pSmart-I-CoV-2 Nsp13 vectors were introduced into *E. coli BL21*(DE3) for protein expression.

#### Proteins expression and purification

After fermentation, the cells were harvested by centrifugation. The supernatant obtained after cell disruption by ultra-sonication was subjected to nickel ion affinity chromatography for purification. The eluted target protein CoV-2 N was further purified by gradient elution using an SP cation exchange column. CoV-2 Nsp13, with a ubiquitin tag, was first purified by nickel ion affinity chromatography, followed by tag removal using SUMO protease. It was then re-purified using a Ni-NTA column. After the ubiquitin tag was removed,CoV-2 Nsp13 was precipitated with ammonium sulfate at a final concentration of 3.5 M, and the protein was re-suspended, filtered, and further purified by size exclusion chromatography using a dextran-based gel filtration column. The final target proteins were analyzed using 12% SDS-PAGE, and both recombinant CoV-2 N and CoV-2 Nsp13 exhibited molecular weights consistent with their theoretical values. The purity of both proteins was greater than 95%.

#### Preparation of substrates

The nucleic acid substrates used in this experiment were synthesized by Sangon Biotech (Shanghai) Co., Ltd. and purified via HPLC (see Table 1). Substrates for EMSA and fluorescence polarization assays were labeled with 3′-6-FAM fluorescein. For the stopped-flow assay, the substrates were labeled with 3′-6-FAM fluorescein, while the complementary single strands were labeled with 5′-HF fluorescein. The substrates for the single-molecule assay were labeled with 5′-biotin.Double-stranded nucleic acid substrates were prepared by annealing complementary single strands in a 1∶1 ratio using a buffer containing 25 mM Tris-HCl (pH 7.6) and 50 mM NaCl. The PCR annealing protocol involved heating at 95°C for 5 min, followed by a gradual decrease in temperature by 1°C every 45 s until reaching 25°C. A 12% native PAGE gel was used for detection.

#### Equilibrium DNA or RNA-binding assay

The binding of CoV-2 N protein or CoV-2 Nsp13 to fluorescein-labeled nucleic acid substrates was analyzed using the fluorescence polarization method with a Spectramax i3X multifunctional plate reader (Molecular Devices, Inc.). Various concentrations of protein were added to a 150 μL solution of buffer A (50 mM NaCl, 25 mM Tris-HCl, pH 7.6) containing 5 nM fluorescein-labeled nucleic acids. Each sample was thoroughly mixed and allowed to equilibrate for 5 min. Measurements were repeated three times per group. The dissociation equilibrium constant (*Kd*) was calculated by fitting the data to Hill’s equation.

$$y=\frac{Pn}{kdn+Pn}$$

where *y* represents the proportion of binding, *P* represents the protein concentration, and *n* represents the Hill’s coefficient, and if *n* > 1 means that more than 1 protein molecule binds to 1 DNA molecule.

#### Electrophoretic Mobility Shift Assay

The unwinding and annealing assays of CoV-2 N were investigated using EMSA (Electrophoretic Mobility Shift Assay). Annealing experiment: Various concentrations of protein were added to two partially complementary single-stranded substrates at a 1∶1 concentration ratio in buffer B (100 mM NaCl, 25 mM Tris-HCl, pH 7.6). The reaction was incubated in a water bath at 30°C for 10 min. Unwinding experiment: Different concentrations of protein were titrated into buffer C (300 mM NaCl, 25 mM Tris-HCl, pH 7.6), containing 10 nM double-stranded nucleic acid substrate. This reaction was also incubated in a water bath at 30°C for 10 min. Immediately after the reaction, proteins were inactivated by adding 2 μL of termination buffer (50 mM EDTA, 2% SDS, 30% glycerol, 0.01% bromophenol blue) on ice. All solutions and samples were pre-cooled on ice for the deconvolution and annealing experiments. The final results were analyzed via 12% Native-PAGE gels electrophoresed at 100 V for 60 min, then detected using a ChemiDoc MP gel imaging system. The results were further analyzed by grayscale scanning with Image Lab software.

#### Stopped-flow FRET measurements

We used a stopped-flow FRET assay to measure the annealing kinetic rate constants of CoV-2 N. The assay was performed using a Bio-Logic SFM-400 mixer and a Bio-Logic MOS450/AF-CD optical system. The annealing reaction was conducted in a two-syringe mixing mode. In channel 1#, a single-stranded substrate labeled with 6-FAM fluorescein was mixed with CoV-2 N, while in channel 2#, a complementary single-stranded substrate labeled with HF fluorescein was injected. Both syringes contained the same annealing reaction buffer B (100 mM NaCl, 25 mM Tris-HCl, pH 7.6). The final concentration of the protein was 600 nM, and the nucleic acid substrate concentration was 4 nM. After the two channels were quickly mixed, the annealing reaction was initiated. During the annealing process, the change in spatial distance between the two fluorescent molecules results in an increase or decrease in the fluorescence signal, allowing the annealing of the two single-stranded nucleic acids to be monitored in real time. The annealing efficiency at time *t* is given by the equation:

$$\eta \left(t\right)=\frac{Fs-F\left(t\right)}{Fs-F100\%}$$

where *F(t)* is the fluorescence signal measured at time *t*, *Fs* is the fluorescence signal at the start of the annealing reaction, and *F*_*100%*_ is the signal obtained from the calibration measurement. The kinetics of DNA annealing efficiency were obtained by normalizing the data curve using this equation.

#### Single-molecule fluorescence resonance energy transfer

The concentration of fixed G4 DNA substrate was 50 pM, and the substrate was incubated in the reaction cell for 10 min. After incubation, free G4 DNA was removed using 200 μL of buffer D (30 mM NaCl, 25 mM Tris-HCl, pH 7.6, 5 mM MgCl_2_, 1 mM DTT). In a specific field of view of an inverted microscope, the donor and acceptor fluorescence of the substrate itself was recorded. After the addition of CoV-2 N, the fluorescence intensity of both the donor and acceptor was recorded again. The fluorescence intensity of the acceptor (*IA*) and the fluorescence intensity of the donor (*ID*) were measured. The structural changes in the G4 DNA were inferred from changes in the FRET efficiency, calculated using the equation: $E=\frac{IA}{IA+ID}$

where *E* represents the FRET transfer efficiency, *IA* is the fluorescence intensity of the acceptor, and *ID* is the fluorescence intensity of the donor.

#### ATPase assay

DNA-dependent ATPase activity was measured using a commercial ATPase assay kit according to the manufacturer’s instructions (Shanghai Beyotime Biotechnology Co., Ltd.). Further details can be found in the Supplementary Materials and Methods.

### Quantification and statistical analysis

#### Data processing and graphing

Data were processed and analyzed using IBM SPSS Statistics 29, and graphing was performed using Origin Pro software.
