# Supplementary material for: The regulatory mechanisms of SARS-CoV-2 N protein helicase and its annealing activity
Source: iScience. 2025 Nov 13;28(12):113983. doi: 10.1016/j.isci.2025.113983 (PMC12702239; doi:10.1016/j.isci.2025.113983)
Supplement: Document S1. Figures S1–S3 [file mmc1.pdf]

iScience, Volume *28*

Supplemental information

The regulatory mechanisms

of SARS-CoV-2 N protein helicase

and its annealing activity

Bo Zhang, Peng Zhou, Zhaoling Lan, Chaoshao Yang, Jida Li, Yi Zhang, Long Gao, Hongyi Wang, Cai Meng, Shizheng Wei, Chenglang Ruan, Yangxue Dai, Yan Xie, and Yang Liu

Supplemental information

Supplementary Figures

Supplementary Figure 1

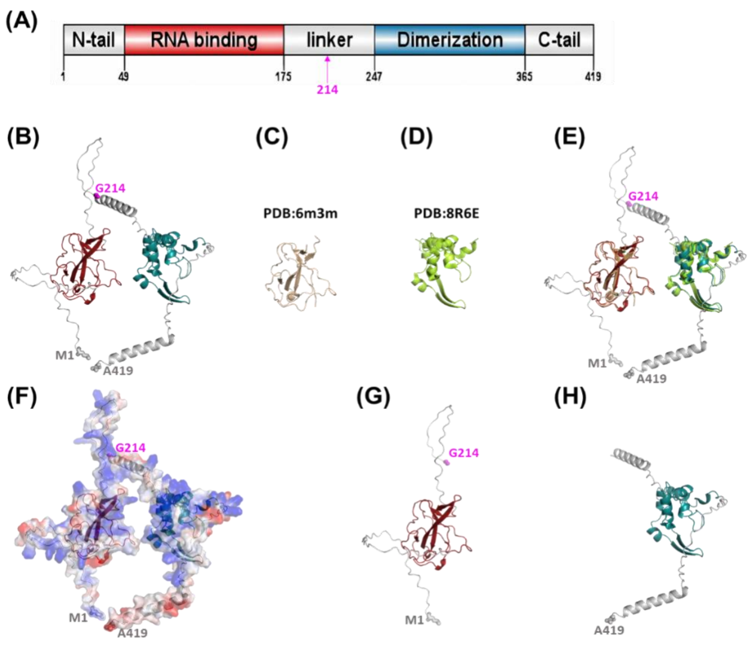

Spplementary Figure 1 Comparative structural analysis of the SARS-CoV-2 N

Note: (A) Schematic representation of the modular organization of the CoV-2 N. (B) The full-length structure of the CoV-2 N was predicted using AlphaFold3. The regions highlighted in firebrick and deepteal represent the RNA-binding domain and the dimerization domain, respectively.(C) Crystal structure of the truncated CoV-2 N (residues 48-173) retrieved from the PDB database (PDB ID: 6M3M). (D) Crystal structure of the truncated CoV-2 N (residues 247-364) retrieved from the PDB database (PDB ID: 8R6E).(E) Superposition of the protein structures shown in panels B, C, and D. (F) Electrostatic potential map of the predicted full-length CoV-2 N structure, where blue represents positively charged regions, red represents negatively charged regions, and white indicates neutral areas.(G and H) Predicted structural models of CoV-2 N truncations designed by our group: CoV-2 N (residues 1-214) in panel G and CoV-2 N (residues 215-419) in panel H.

Supplementary Figure 2

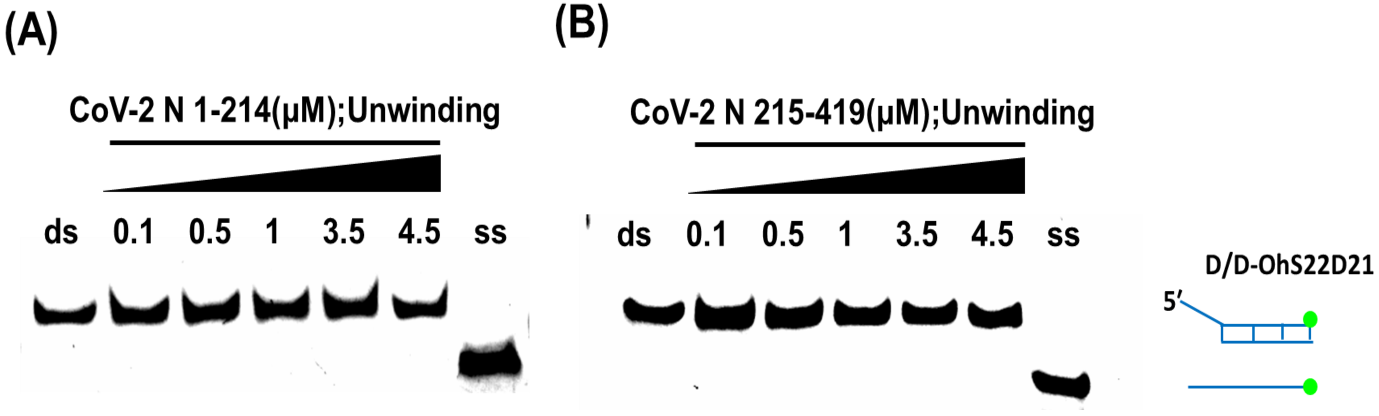

Supplementary Figure 2   Helicase activity assay of CoV-2 N truncated proteins

Note: (A) CoV-2 N (residues 1–214).(B) CoV-2 N (residues 215–419).Lanes labeled “ds” represent the double-stranded DNA substrate (5′D/D-OhS22D21), while lanes labeled “ss” represent the FAM-labeled single-stranded DNA control (3′-FAM-S43).All DNA substrates were used at a final concentration of 10 nM, with increasing concentrations of protein applied.

Supplementary Figure 3

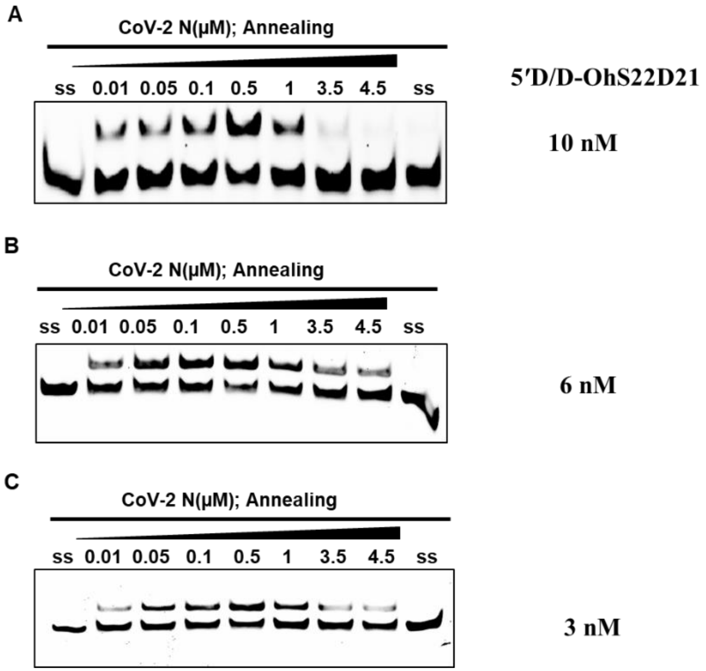

Supplementary Figure 3 Effect of different DNA substrate concentrations on CoV-2 N annealing activity

Note: A concentration of 0.5 μM CoV-2 N represents the critical threshold for inhibiting annealing activity, independent of the DNA substrate ratio. (A) Substrate concentration: 10 nM; (B) 6 nM; (C) 3 nM. Lanes labeled “ds” correspond to the double-stranded DNA substrate (5′D/D-OhS22D21), while lanes labeled “ss” correspond to the FAM-labeled single-stranded DNA control (3′-FAM-S43). Substrates were used at the concentrations indicated in the figure, with increasing protein concentrations. All experiments were performed under the conditions described in the “Materials and Methods” section.
